# Identifying Key Regulators of Keratinization in Lung Squamous Cell Cancer Using Integrated TCGA Analysis

**DOI:** 10.3390/cancers15072066

**Published:** 2023-03-30

**Authors:** Yusri Dwi Heryanto, Seiya Imoto

**Affiliations:** 1 Division of Health Medical Intelligence, Human Genome Center, The Institute of Medical Science, The University of Tokyo, 4-6-1 Shirokanedai, Minato-ku, Tokyo 108-8639, Japan; 2 Laboratory of Sequence Analysis, Human Genome Center, The Institute of Medical Science, The University of Tokyo, 4-6-1 Shirokanedai, Minato-ku, Tokyo 108-8639, Japan

**Keywords:** lung cancer, keratin, gene set score, transcriptome analysis, miRNA analysis, methylation analysis

## Abstract

**Simple Summary:**

Keratins are the intermediate filament-forming proteins of epithelial cells that support cell structure and tissue homeostasis. In lung squamous cell cancer, keratins are frequently used as diagnostic tumor markers. However, several studies have shown evidence of keratin’s roles in cancer invasion, metastasis, and therapy resistance. In this study, we aimed to identify key regulators of the cancer-related keratinization process in LUSC. Our findings may help others to gain insight into the cancer-related keratinization process and to find potential targets for diagnostics and therapy for LUSC.

**Abstract:**

Keratinization is one of lung squamous cell cancer’s (LUSC) hallmark histopathology features. Epithelial cells produce keratin to protect their integrity from external harmful substances. In addition to their roles as cell protectors, recent studies have shown that keratins have important roles in regulating either normal cell or tumor cell functions. The objective of this study is to identify the genes and microRNAs (miRNAs) that act as key regulators of the keratinization process in LUSC. To address this goal, we classified LUSC samples from GDC-TCGA databases based on their keratinization molecular signatures. Then, we performed differential analyses of genes, methylation, and miRNA expression between high keratinization and low keratinization samples. By reconstruction and analysis of the differentially expressed genes (DEGs) network, we found that *TP63* and *SOX2* were the hub genes that were highly connected to other genes and displayed significant correlations with several keratin genes. Methylation analysis showed that the *P63*, *P73*, and *P53* DNA-binding motif sites were significantly enriched for differentially methylated probes. We identified *SNAI2, GRHL3, TP63, ZNF750*, and *FOXE1* as the top transcription factors associated with these binding sites. Finally, we identified 12 miRNAs that influence the keratinization process by using miRNA–mRNA correlation analysis.

## 1. Introduction

Lung squamous cell carcinoma (LUSC) accounts for 20% of all lung cancer diagnoses and is the second most common subtype of lung cancer [1]. One of the key histological features of LUSC is keratinization [2]. When epithelial cells are keratinized to form a keratin layer, a unique program of terminal differentiation and apoptotic cell death follows [3]. Keratinization is an example of epithelial cell adaptation to protect cell integrity from environmental influences, such as physical damage, infection, or xenobiotic substances [4]. In addition to their role as epithelial cell protectors, keratins have other important roles as cellular function regulators, such as apical–basal polarization, cell size determination, protein translation, and organelle position regulation [5].

In cancer, keratins have been used extensively as immunohistochemical markers [6]. Beyond their role as tumor markers, keratins have active roles in cancer cell growth, migration, and invasion [5,7]. In LUSC, keratinization is strongly associated with smoking [8]. This is not surprising because keratinization is one of the frontline defense systems that interact with external harmful substances. Despite the importance of keratin’s roles in healthy cells and cancer cells, insights into keratinization mechanisms and regulations remain incomplete.

This study is an extension of our previous work that investigated the gene-methylation regulation network in LUSC [9]. In our prior investigation, we discovered that the genes responsible for the keratinization process formed the second largest community, following the genes related to the cell cycle in the LUSC gene regulation network. Moreover, the keratinization gene community exhibited close interactions with other processes, such as detoxification and the cell cycle, suggesting that keratinization is heavily dysregulated in LUSC and plays a significant role in cancer pathogenesis. However, we did not thoroughly examine the primary regulators of keratinization in our earlier work.

Therefore, this study aimed to identify the key regulators of the cancer-related keratinization process in LUSC. To address this goal, we performed an integrative analysis of the transcriptomic, methylomic, and miRNA LUSC profiles from the Genome Data Common—The Cancer Genome Atlas (GDC-TCGA) LUSC database. We categorized the samples based on their keratinization signature scores. Then, we compared the groups using three different workflows: the differentially expressed gene network [9], Enhancer Linking by Methylation/Expression Relationships (ELMER) [10], and mirTarRNASeq [11] to identify the important genes, binding motifs, transcription factors, and miRNAs involved in the LUSC keratinization process. The results of our study may provide a basis for the identification of novel biomarkers and facilitate a deeper understanding of the LUSC keratinization process.

## 2. Materials and Methods

### 2.1. Overview

**Figure 1:**
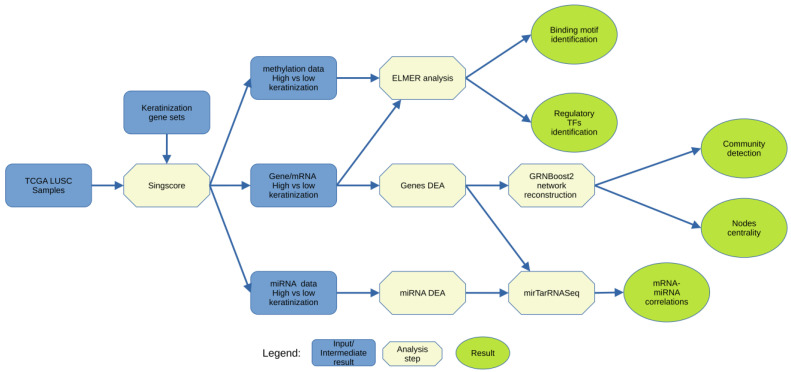
Overall pipeline of the analysis in our study. We divided the LUSC samples using single-sample gene set scoring, Singscore, with keratinization-related gene sets. Then, we performed differential expression analyses (DEA) of gene, methylation, and miRNA expression between high keratinization and low keratinization groups. Differentially expressed gene networks, ELMER, and mirTarRNASeq analysis were performed to identify key regulatory genes, transcription factors, and miRNA.

The overall pipeline in our study is depicted in Figure 1. First, we downloaded and analyzed the messenger RNA (mRNA), methylation, and microRNA (miRNA) profiles from the GDC-TCGA LUSC database. We utilized the Singscore individual sample gene set scoring approach [12] for classifying the samples into three groups (low, medium, and high keratinization) based on their keratinization signature scores. Then, we performed differential expression analyses (DEA) of the transcriptomic, methylomic, and miRNA profiles of these groups. Finally, we identified the key regulatory genes, transcription factors (TFs), and miRNAs using network analysis [9,13], the ELMER algorithm [14], and miRNA–mRNA correlation analysis [11], respectively.

### 2.2. Data Acquisition and Preparation

The gene expression, methylation beta value, and miRNA isoform data were obtained from the GDC-TCGA harmonized database using the Bioconductor package TCGAbiolinks [15]. The gene expression data consisted of 502 LUSC primary tumor samples and 49 normal tissue samples. Then, we paired the samples from the methylation and miRNA expression data with their corresponding samples from the gene expression data. We removed the data that have no corresponding paired samples in gene expression data. Finally, we only kept the primary tumor samples’ methylation and miRNA expression data.

Next, we filtered non-relevant data from gene, methylation, and miRNA data for further analysis. The genes with low counts across most samples were discarded from the analysis. Here, we retained genes that had a transcript count per million >1 across over 50% of the samples. We discarded the genes that had identical gene names to enforce unique mapping. For methylation data, we removed the probes with at least one missing value and removed the probes in chromosomes X, Y, and NA. We used only mature miRNA expression data for miRNA analysis.

### 2.3. Data Single-Sample Scoring and Clustering

We downloaded the Gene Ontology (GO) gene sets from the GSEA database (https://www.gsea-msigdb.org accessed on 6 November 2022) and selected the gene sets related to the keratinization process or keratinocyte differentiation/development. The gene sets and their brief descriptions are listed in Table 1.

We used the Singscore method to score individual samples against these 10 keratinization-related gene sets [12]. In Singscore methods, genes are sorted according to their transcript levels, with upregulated sets being ranked in ascending order and downregulated sets in descending order. Subsequently, rank-based statistics are utilized to determine the scores for each individual sample [12]. Hierarchical clustering with complete linkage was performed to divide the samples into subgroups with different degrees of keratinization according to the keratinization signature scores. The optimal number of clusters was determined using the R package NbClust, which provides 30 indices that determine the number of clusters.

### 2.4. Differential Expression Analysis (DEA) of the Genes

We performed DEA of the tumor samples in high keratinization versus low keratinization groups for identifying key regulators of the keratinization process in LUSC. We preprocessed the gene expression data using the TCGAbiolinks package and workflow from Silva et al. [16]. In short, we removed outliers, failed hybridization, or mistracked samples by performing Array–Array Intensity Correlation using the TCGAanalyze_Preprocessing function. Next, we normalized mRNA transcript samples using the TCGAanalyze_Normalization, which encompasses the functions of the EDASeq package. Finally, we filtered genes with low signals across samples using TCGAanalyze_Filtering functions. The function TCGAanalyze_DEA was applied to identify the differentially expressed genes (DEG) between high keratinization and low keratinization groups. We defined the genes with the absolute log fold change ≥1 and FDR < 0.01 as the significant DEGs.

### 2.5. DEGs Network Reconstruction and Analysis

We modified the workflow from our previous work to perform DEGs network reconstruction and analysis [9]. In summary, we performed log(1 + x) transformation to the significant DEG’s expression matrix before we inputted it into the network reconstruction algorithm. We used the GRNBoost2 algorithm implemented in the Python package Arboreto to reconstruct the DEG regulatory network [17]. Briefly, this algorithm involves training a gradient boosting machine model for each differentially expressed gene in the dataset to predict its expression profile using the expression values of a set of candidate transcription factors (TFs). Each model produces a partial gene regulation network with regulatory associations from the best-predicting TFs to the target gene. All regulatory associations are combined and sorted by importance to finalize the regulatory network output.

Community detection of the network was performed using the Leiden algorithm implemented in the Python package leidenalg [18]. The community Reactome pathway-based analysis was performed using g:Profiler [19]. Then, we calculated the node betweenness centrality and degree using the python-igraph package. We performed Pearson’s correlation test between the expression of the genes with high centrality or high out-degree index and the keratin genes. We adjusted the *p*-value using Bonferroni correction and considered an adjusted *p*-value < 0.05 as significant.

### 2.6. Methylation Motif and Regulatory Transcription Factor Identification

First, we compared the mean DNA methylation levels across the three groups. We performed ANOVA test to determine if there was any difference between the means of different groups. We considered ANOVA test *p*-value < 0.05 as significant.

Next, we used ELMER analysis workflow to identify the enriched motifs for the probes that were significantly differentially hypomethylated in the high keratinization samples relative to low keratinization samples [10]. ELMER workflow consists of 5 main steps: (1) identifying distal probes on Infinium Human Methylation 450K arrays, (2) selecting significantly hypomethylated probes by comparing the methylation level of each probe for all samples in group 1 compared to all samples in group 2, using an unpaired one-tailed *t*-test, (3) identifying putative target genes for differentially methylated probes by performing Mann–Whitney U test for each candidate probe–gene pair, (4) identifying enriched motifs of hypomethylated probes using Fischer’s exact test, and (5) identifying regulatory transcription factors (TFs) whose expression is associated with TF binding motif DNA methylation by performing Mann–Whitney U test for each candidate motif–TF pair. A motif was considered significantly enriched if the 95% confidence interval of the odds ratio was greater than 1.1. The regulatory TFs were ranked by their *p*-value and those in the top 5% of the smallest *p*-value were considered candidates for upstream regulators.

### 2.7. miRNA–mRNA Relationships Analysis

We used mature miRNA count data to perform DEA of miRNA by TCGAbiolinks package. We filtered the miRNAs with low signals across samples using TCGAanalyze_Filtering functions. The function TCGAanalyze_DEA was applied to identify the differentially expressed miRNA between high keratinization and low keratinization groups. The absolute log fold change ≥1 and FDR < 0.01 were used as the thresholds to select significantly differentially expressed miRNAs. We inputted the log fold change of significant differentially expressed genes from previous DEG analysis and miRNAs to the R Bioconductor package mirTarRnaSeq to identify some significant miRNA–mRNA correlations [11]. The mirTarRnaSeq approach estimated the difference between the miRNA–mRNA fold change, followed by the generation of a background distribution that represents random differences in fold chance. Then, it ranked the difference values against the background distribution to obtain the *p*-value, FDR, and test statistics estimates. Finally, we intersected our significant miRNA–mRNA correlations result with miRanda database binding predictions [20].

### 2.8. Source Code

The complete source code and the parameter details needed to reproduce our study have been stored in the public repository (https://github.com/yusri-dh/Keratinization_LUSC accessed on 19 December 2022).

## 3. Results

### 3.1. Hierarchical Clustering Based on Single-Sample Scoring against Keratinization-Related Gene Set Identifies Three LUSC Phenotypes

**Figure 2:**
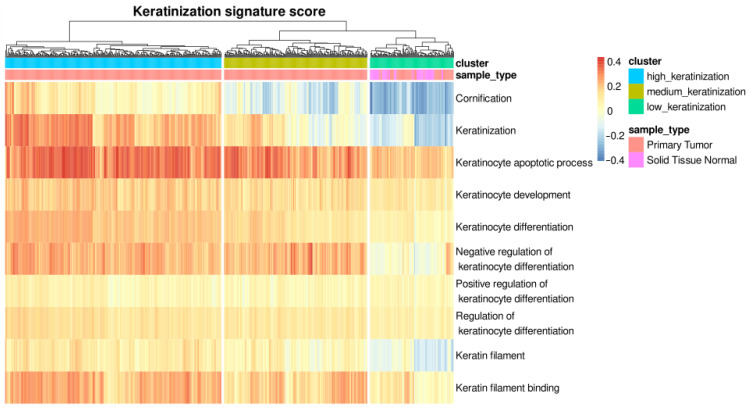
The classification of the LUSC samples into three groups, low keratinization, medium keratinization, and high keratinization, based on the keratinization-related GO gene sets. Each column represents a sample. Each row represents a keratinization molecular signature from Gene Ontology. The heatmap units are the Singscore value. Positive Singscore values represent enrichment of the gene set. In contrast, negative values represent underrepresentation of the gene sets.

Based on the single-sample scores against the 10 keratinization-related gene sets, we hierarchically clustered LUSC samples into three groups: high keratinization, medium keratinization, and low keratinization (Figure 2). We selected K = 3 as the optimal number of clusters based on the result of the NbClust analysis. The high keratinization group was characterized by the enrichment in cornification and keratinization gene sets. In contrast, low keratinization samples have an inverse enrichment in cornification and keratinization gene sets. They also have a lower expression of genes related to keratinocyte differentiation and the apoptotic process than samples in the high keratinization group. All normal samples were in the low keratinization group. We compared the samples in high and low keratinization samples in subsequent analyses for identifying key regulators of the keratinization process in LUSC.

### 3.2. DEGs Network Reconstruction and Analysis

**Figure 3:**
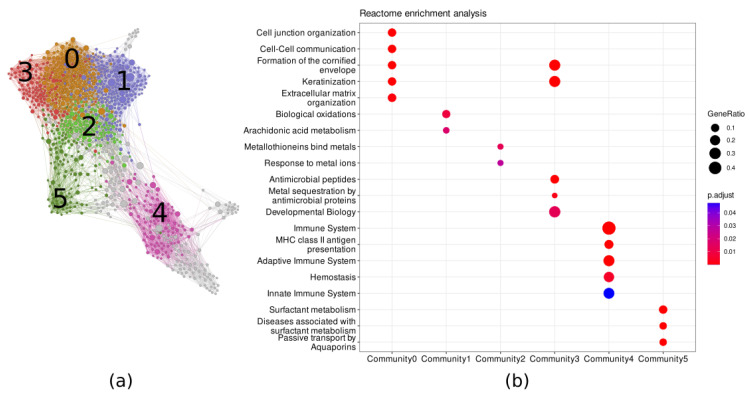
The gene regulation network of the DEGs between high keratinization and low keratinization samples. (**a**) The gene regulation network reconstructed by GRNBoost2 algorithm. Each community was labeled with its community number and different color. (**b**) The Reactome pathway-based enrichment analysis showed the functional classes of each community that connected to the keratinization process in LUSC.

We identified 1287 significant DEGs (FDR-adjusted *p* < 0.01) by comparing the mRNA expression profiles between the high keratinization and low keratinization groups (Table S1). To gain a deeper understanding of the functions and interactions of DEGs, we used the GRNBoost2 network reconstruction implemented in the Arboreto package [17]. The inferred network had 1097 nodes and 9145 edges (Figure 3a). The nodes’ properties and edge list are provided in Tables S2 and S3, respectively. Using the Leiden algorithm, we identified 10 major communities in the keratinization DEGs network. We named the largest community Community 0, the second largest Community 1, and so on. The Reactome pathway-based enrichment analysis showed that Community 0 and 3 mainly included keratinization-related genes. Community 1 and 2 networks were enriched in biological oxidation and detoxification-related genes; Community 4 contained immune system genes; and Community 5 contained genes that were responsible for surfactant metabolism (Figure 3b). From the centrality analysis, we found that *SOX2, FOXE1,* and *SYNE1* were the genes with the highest betweenness centrality (Table 2). Meanwhile, *TP63, CD53*, and *NCKAP1L* were the genes with the highest node out-degree index (Table 2). We selected the genes with the highest betweenness centrality and out-degree index to perform Pearson’s correlation test with keratin genes. The TP63 genes had a strong and significant positive correlation (Pearson’s R > 0.5, adjusted *p* < 0.05) with several keratin genes: *KRT5, KRT6A, KRT6B, KRT13, KRT14, KRT15, KRT16,* and *KRT17* (Figure S1a). Meanwhile, the *SOX2* gene had a medium and significant positive correlation (Pearson’s R > 0.3, adjusted *p* < 0.05) with *KRT5, KRT6A, KRT6B, KRT13, KRT15*, and *KRT19* (Figure S1b).

### 3.3. P63, P73, and P53 Were the Top Three Enriched Motifs for Hypomethylated Probes

**Figure 4:**
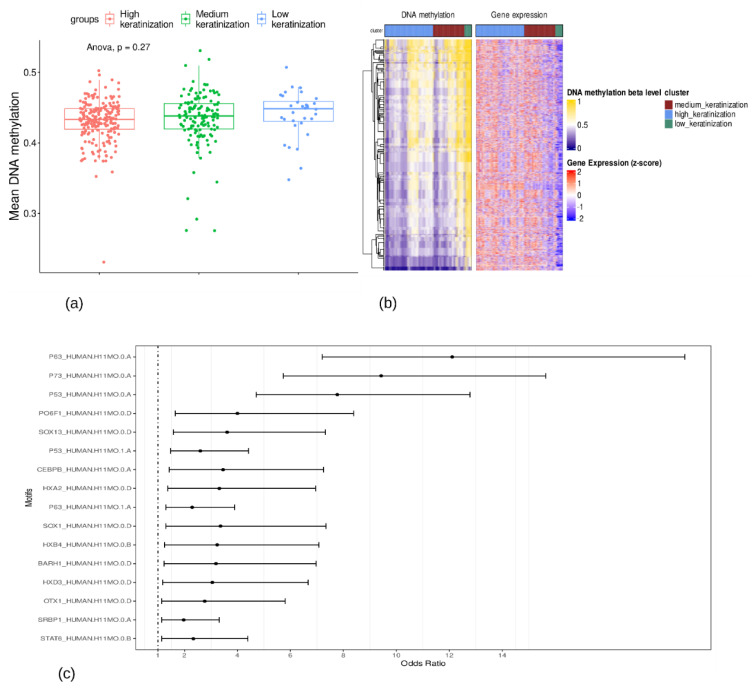
The methylation analysis of LUSC tumor samples. (**a**) The mean methylation status across three groups. (**b**) The methylation levels of the significant gene-probe pairs. The rows and columns in DNA methylation heatmap represent the probes and samples, respectively. The rows and columns in gene expression heatmap represent the corresponding genes and samples. (**c**) The enriched binding motifs for the probes that are significantly hypomethylated in high keratinization samples relative to low keratinization samples.

Through ANOVA analysis, we found no significant differences in overall DNA methylation levels across the three groups (Figure 4a). Then, using an ELMER analysis, we found 284 gene–probe pairs that were significantly hypomethylated in high keratinization samples with FDR-adjusted *p* < 0.001. Figure 4b displays the methylation levels of the identified significant probes, along with the expression patterns of the corresponding gene pairs. A list of all significant gene–probe pairs and their *p*-values is given in Table S4. From all significant pairs, we could identify enriched motifs for the probes that are significantly hypomethylated in high keratinization samples relative to low keratinization samples (Figure 4c). Then, ELMER identified the master regulator TFs corresponding to each of the motifs enriched in the previous analysis step. From all enriched motifs, we could only obtain the significant regulatory TFs associated with the top three binding motifs: the *P63*, *P73*, and *P53* motifs. The top 5% of TFs associated with the binding motifs are listed in Table 2.

### 3.4. The miRNA–mRNA Relationships Analysis

**Figure 5:**
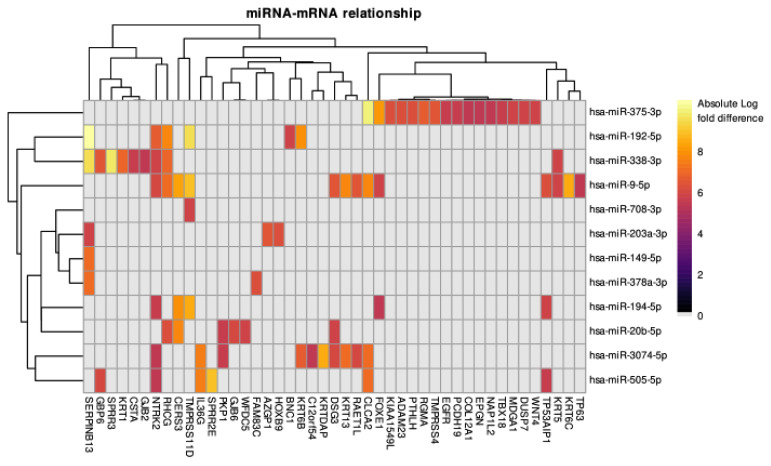
High keratinization vs. low keratinization groups heatmap of all significant miRNA–mRNA interrelations predicted by both mirTarRnaSeq and miRanda. Gray color represents no significant interrelation identified by mirTarRnaSeq. The heatmap units are the absolute difference between miRNA and mRNA fold changes.

The mirTarRnaSeq results showed that there were 913 miRNA–mRNA pairs that had a significant relationship (FDR-adjusted *p* < 0.05). From these 913 miRNA–mRNA pairs, there were 78 pairs that intersected with the miRanda database findings. Figure 5 shows these miRNA–gene pairs and their relationship strength in the unit of absolute log fold difference. Out of all the miRNAs, hsa-miR-375-3p and hsa-miR-9-5p had the greatest number of pairs (16 and 13 pairs, respectively). Whereas, *NRTK2* and *SERPINB13* were the genes that were affected by the highest number of miRNAs. The regulatory miRNAs are listed in Table 2.

## 4. Discussion

Several methods for scoring individual samples against gene sets have been developed, including ssGSEA (single-sample gene set enrichment analysis) [21], GSVA (gene set variation analysis) [22], and Singscore [12]. These frameworks have been used for dimensional reduction, clustering, and condensing information from transcriptomic data [23,24,25]. In this study, we used Singscore to classify LUSC samples into three groups (i.e., low, medium, and high keratinization) based on their keratinization signature scores. The high keratinization group demonstrated the upregulation of genes involved in the cornification, keratinization, and keratinocyte apoptotic processes, whereas these gene sets were underrepresented in the low keratinization group. Using differential analyses of high and low keratinization groups, we investigated the genes, transcription factors, and miRNA that act as key regulators of the LUSC keratinization process.

To identify keratinization core regulatory genes, we employed the same network analysis approach utilized in our prior research [9]. In this approach, we inferred and reconstructed the DEGs network, which provided us with a blueprint of the gene–gene interactions in cancer. Here, we calculated the nodes’ out-degree index and betweenness centrality to investigate the roles of some nodes and their impact on the networks. The out-degree index of a node is the number of edges that are going out from the node. Narang et al. showed that the important TFs have a high out-degree index, implying that TFs usually target a large number of genes [13]. In our study, *TP63* had the highest out-degree index. *TP63* is highly expressed in the basal compartment of the lung airways and is required for progenitor cell development, epidermal stratification, and to maintain the proliferative potential of basal keratinocytes during homeostasis [26,27,28]. In the gene regulation network, the most important nodes are not always the ones with the most edges, but the ones that connect groups or have the most control over the flow of information. The betweenness centrality measures the number of times a node lies on the shortest path between other nodes. This measure shows which nodes act as bridges between nodes and have significant control over the information flow in the gene regulatory network [9,13]. We found *SOX2* to be the gene with the highest betweenness centrality. Moreover, it is also in the top six highest out-degree genes, indicating that *SOX2* has important roles in the LUSC keratinization process. *SOX2* is a transcription factor that promotes the development and maintenance of squamous epithelium and is an essential regulator of pluripotent stem cells [29]. Because of their adjacent chromosomal localization (3q), *SOX2* and *TP63* are frequently co-amplified in cancer [29]. *TP63* and *SOX2* collaborate to regulate multiple genes involved in squamous carcinogenesis [29]. Our study found that *TP63* and *SOX2* had significant medium and strong correlations with several keratin genes, such as *KRT5, KRT6A, KRT6B, KRT13, KRT14, KRT15,* and *KRT17*. This finding indicated that *TP63* and *SOX2* have a direct influence on the keratinization process by modulating keratinization genes in LUSC.

The community gene set enrichment analysis of the DEGs network can reveal the important gene interactions and processes that influence keratinization. We found that the genes related to detoxification (i.e., biological oxidations and metallothioneins binding to metals), the immune system, and surfactant metabolism were connected with the keratinization process in LUSC. Lung epithelial cells are one of the first lines of defense that interact directly with potentially harmful substances. Smoking, as the primary LUSC risk factor, may be responsible for the detoxification–keratinization relationship [8]. Some studies have highlighted the roles of keratins as regulators of inflammation and immunity in epithelia [30,31,32,33]. The keratin 76 downregulation enhanced the accumulation of T regulatory cells, leading to a drop in the anti-tumor response in oral squamous cell carcinoma [33]. In basal cell carcinoma, the genetic ablation of keratin 17 decreased inflammation and polarized the inflammatory response towards T-helper-2-cells. However, the detailed mechanism of how keratinization and the immune system are interrelated in LUSC remains poorly understood. To the authors’ knowledge, no papers have investigated the direct relationship between keratinization and surfactant. In the lung, the surfactant has important roles as a regulator of the immune system and homeostasis [34,35]. Further study is necessary to elucidate the relationship between keratinization and surfactant metabolism.

In addition to genetic changes, many DNA methylation alterations are associated with the LUSC pathophysiology process. We found that the *P63*, *P73*, and *P53* binding motifs were enriched for hypomethylated probes in high keratinization samples. *P73* and *P63* are two homologs of *P53* and have a high degree of structural symmetry with *P53*. Thus, *P73* and *P63* can bind to most of the p53-responsive binding sites, and vice versa [36]. *SNAI2, GRHL3, TP63, ZNF750*, and *FOXE1* were the top TFs associated with the *P63*, *P73*, and *P53* binding motifs in our study. These transcription factors have important roles in epithelial differentiation and keratinization [37,38,39,40,41].

MicroRNAs, or miRNAs, are small non-coding RNAs that play important roles in post-transcriptional gene regulation [42,43]. These miRNAs may directly regulate keratin proteins. For example, hsa-miR-3074-5p, which targets *KRT13* and *KRT6B*, or hsa-miR-9-5p, which targets *KRT6C*, *KRT13*, and *KRT5*. Other miRNAs regulate keratinization-related processes, such as hsa-miR-149-5p, hsa-miR-192-5p, and hsa-miR-378a-3p, which target *SERPINB13*, or hsa-miR-3074-5p, which target *KRTDAP*. Downregulation of the *SERPINB13* protein was significantly associated with keratinocyte/epithelial cell differentiation and apoptosis [44]. Meanwhile, *KRTDAP* is a gene that plays important roles in the epithelium stratification process [45]. In our analysis, we found that hsa-miR-375-3p and hsa-miR-9-5p had the greatest number of pairs with other DEGs. This suggests that the regulation of the LUSC keratinization process is vastly influenced by hsa-miR-375-3p and hsa-miR-9-5p. The expression of hsa-miR-375-3p has been shown to have potential as a promising diagnostic marker in oral cancer [46], breast cancer [47], and head and neck cancer [48]. Additionally, a study showed that hsa-miR-375-3p may have a suppressor role in bladder cancer via the Wnt/beta-catenin pathway [49]. In the case of hsa-miR-9-5p, some studies have demonstrated that hsa-miR-9-5p has potential as a biomarker of therapy response in nasopharyngeal carcinoma [50], cervical cancer [51], and leukemia [52]. To the best of the authors’ knowledge, there have been no experimental studies conducted to explore the involvement of hsa-miR-375-3p and hsa-miR-9-5p in the keratinization process or to evaluate their potentials as diagnostic biomarkers, making it an interesting subject for future studies.

## 5. Conclusions

The single-sample scoring approach was used in our study to classify the sample into three groups based on their keratinization signatures. Through a comparison of high keratinization versus low keratinization samples using DEA, we were able to identify several transcription factors (TFs), binding motifs, and miRNAs that are likely involved in regulating the keratinization process of lung squamous cell carcinoma (LUSC). Specifically, we emphasized the importance of the *TP63* and *SOX2* genes, the *P63*, *P73*, and *P53* DNA binding motifs, and the hsa-miR-375-3p and hsa-miR-9-5p miRNAs as potential key regulators of keratinization in LUSC. Further studies of these regulators might help researchers acquire a deeper understanding of the keratinization process in LUSC and find potential novel biomarkers.

## Figures and Tables

**Table 1:** Keratinization GO gene sets and their description.

GO Subset	Name	GO Description
Biological Process	KERATINIZATION	The process in which the cytoplasm of the outermost cells of the vertebrate epidermis is replaced by keratin.
Biological Process	CORNIFICATION	A unique type of programmed cell death that leads to the formation of keratin layer.
Biological Process	KERATINOCYTE APOPTOTIC PROCESS	Any apoptotic process in a keratinocyte.
Biological Process	KERATINOCYTE DEVELOPMENT	The process whose specific outcome is the progression of a keratinocyte over time, from its formation to the mature structure.
Biological Process	KERATINOCYTE DIFFERENTIATION	The process in which a relatively unspecialized cell acquires specialized features of a keratinocyte.
Biological Process	NEGATIVE_REGULATION OF KERATINOCYTE DIFFERENTIATION	Any process that stops, prevents, or reduces the frequency, rate, or extent of keratinocyte differentiation.
Biological Process	POSITIVE REGULATION OF KERATINOCYTE DIFFERENTIATION	Any process that activates or increases the frequency, rate, or extent of keratinocyte differentiation.
Biological Process	REGULATION OF KERATINOCYTE DIFFERENTIATION	Any process that modulates the frequency, rate, or extent of keratinocyte differentiation.
Cellular Component	KERATIN FILAMENT	A filament composed of acidic and basic keratins (types I and II), typically expressed in epithelial cells.
Molecular Function	KERATIN FILAMENT BINDING	Binding to a keratin filament.

**Table 2:** The top identified regulatory factors that influence keratinization process in LUSC.

Top 20 genes ranked by betweenness centrality	*SOX2*, *FOXE1*, *SYNE1*, *SPIB*, *JMJD7-PLA2G4B*, *FER1L4*, *DLX6*, *ARNT2*, *GNG11*, *VAMP5*, *ABCC5*, *ICAM1*, *ACSL5*, *TREM1*, *PKP1*, *TSPAN18*, *COL7A1*, *MICAL1*, *ANKRD36BP2*, *CXCL1*
Top 20 genes ranked by nodes out-degree index	*TP63*, *CD53*, *NCKAP1L*, *KRT6A*, *PKP1*, *SOX2*, *PTPRC*, *NTRK2*, *CD3E*, *GBP6*, *CD2*, *HLA-DMB*, *GIMAP4*, *SASH3*, *GJB5*, *FAT2*, *CLCA2*, *ITK*, *DOCK2*, *ABCC5*
Top 5% TFs related to hypomethylated probes	*SNAI2*, *GRHL3*, *TP63*, *ZNF750*, *FOXE1*, *IRF6*, *BNC1*, *ZNF385A*, *PITX1*, *HES2*, *KLF5*, *SOX15*, *FOXN1*, *HOMEZ*, *OVOL1*, *NFE2L2*, *ZBTB7C*, *GRHL1*, *RARG*, *ZNF488*, *SOX2*, *ARNTL2*, *KLF3*, *DLX5*, *IRX4*, *SOX21*, *YBX3*, *BCL11B*, *ZNF365*, *RAG1*, *PPARA*, *TCF20*, *TBX18*, *MAF*, *EEA1*, *TSHZ2*, *HOXA1*, *FLYWCH1*, *HOXD11*, *TEF*, *ZIC5*, *DMRT2*, *NR1D1*, *ZBTB7A*, *FEZF1*, *HOXD10*, *FOXD1*, *MXD1*, *ZNF703*, *ELF4*, *KLF4*, *FOXQ1*, *TP73*, *HES1*, *GLI2*, *PRRX2*, *AEBP2*, *SHOX2*, *TFAP2C*, *FOXF2*
Significant regulatory miRNA	hsa-miR-20b-5p, hsa-miR-3074-5p, hsa-miR-375-3p, hsa-miR-194-5p, hsa-miR-505-5p, hsa-miR-9-5p, hsa-miR-338-3p, hsa-miR-378a-3p, hsa-miR-192-5p, hsa-miR-708-3p, hsa-miR-203a-3p, hsa-miR-149-5p

## Data Availability

All of the data can be downloaded from Genome Data Common—The Cancer Genome Atlas (GDC-TCGA) LUSC database. The complete source code and the parameter details to reproduce our study have been stored in a public repository (https://github.com/yusri-dh/Keratinization_LUSC accessed on 19 December 2022).

## Supplementary Materials

The following supporting information can be downloaded at: https://www.mdpi.com/article/10.3390/cancers15072066/s1, Table S1: The list of significant DEGs between high keratinization group and low keratinization group. Table S2: The node properties of DEGs network. Table S3: The edge list of DEGs network. Table S4: The significantly hypomethylated gene–probe pairs in tumor samples with high keratinization. Figure S1: Pearson’s correlation between the TP63/SOX2 and keratins genes.
