# Supplementary material for: Identifying Key Regulators of Keratinization in Lung Squamous Cell Cancer Using Integrated TCGA Analysis
Source: Cancers (Basel). 2023 Mar 30;15(7):2066. doi: 10.3390/cancers15072066 (PMC10092975; doi:10.3390/cancers15072066)
Supplement: Supplementary file 1 [file cancers-15-02066-s001.zip › Figure S1 Correlation between TP63 and SOX2 and keratin genes.pdf]

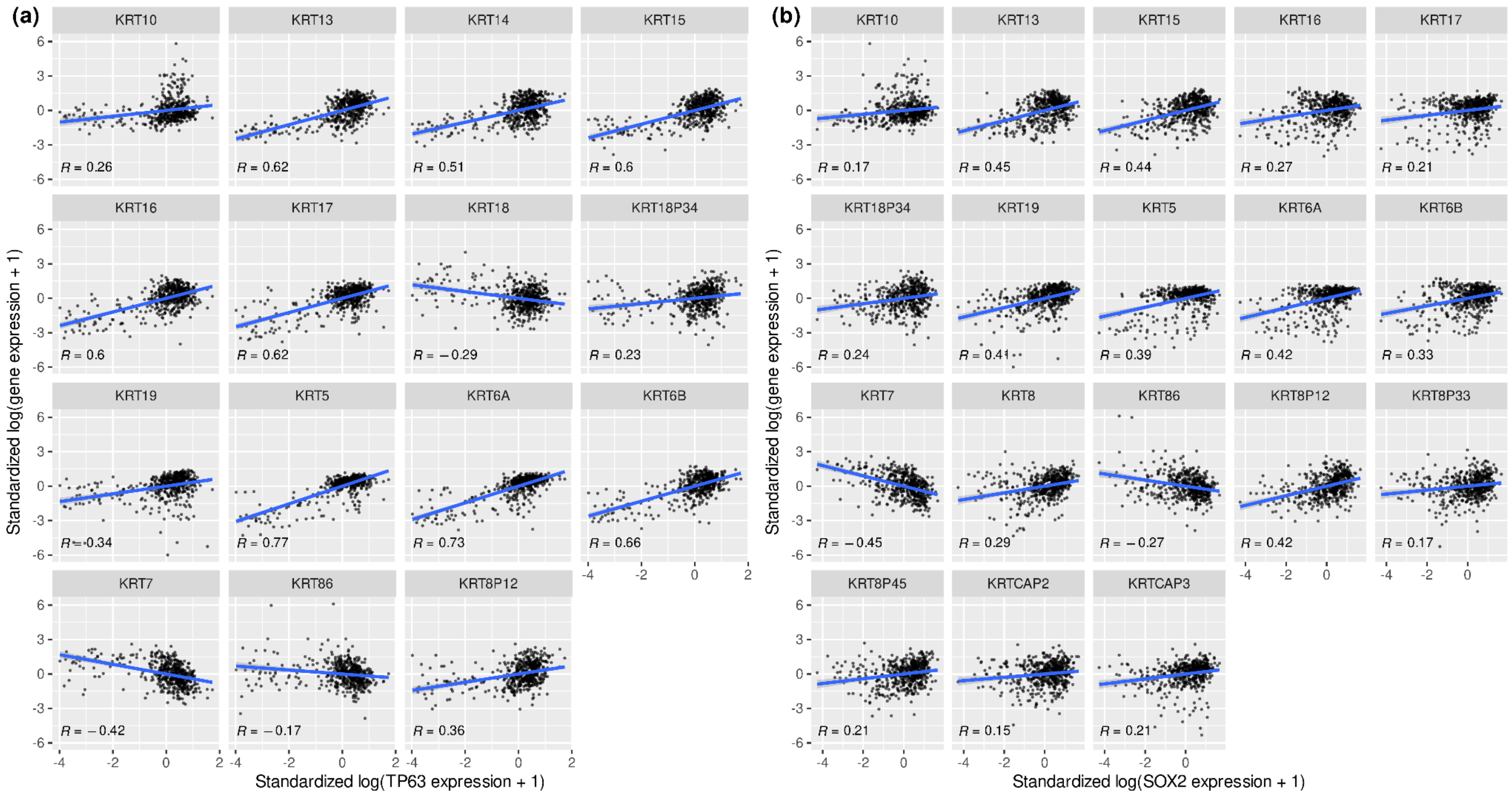

Figure S1. The Pearson correlation between the TP63/SOX2 and keratins genes. (a) TP63 was the genes with the highest out-degree index. Meanwhile, (b) SOX2 was the genes with the highest betweenness centrality. We adjusted the P value of the correlation test using Bonferroni correction. All of the correlations depicted in this figure have adjusted-P < 0.05.
